# ICARUS—Very Low Power Satellite-Based IoT

**DOI:** 10.3390/s22176329

**Published:** 2022-08-23

**Authors:** Marco Krondorf, Steffen Bittner, Dirk Plettemeier, Andreas Knopp, Martin Wikelski

**Affiliations:** 1 Faculty of Engineering, HTWK Leipzig, University of Applied Sciences, 04277 Leipzig, Germany; 2 INRADIOS Rohde & Schwarz GmbH, 01187 Dresden, Germany; 3 Photonics and Antenna Design, TU Dresden, 01062 Dresden, Germany; 4 Institute of Information Technology, University of the Bundeswehr Munich, 85579 Neubiberg, Germany; 5 Space Systems Academic Group, Naval Postgraduate School, Monterey, CA 93943, USA; 6 Department of Migration, Max Planck Institute of Animal Behavior, 78315 Radolfzell, Germany; 7 Centre for the Advanced Study of Collective Behaviour, University of Konstanz, 78464 Konstanz, Germany

**Keywords:** satellite IoT, very-low-power signaling, CDMA, random access

## Abstract

The ICARUS (International Cooperation for Animal Research Using Space) satellite IoT system was launched in 2020 to observe the life of animals on Earth: their migratory routes, living conditions, and causes of death. These findings will aid species conservation, protect ecosystem services by animals, measure weather and climate, and help forecast the spread of infectious zoonotic diseases and possibly natural disasters. The aim of this article is to explain the system design of ICARUS. Essential components are ‘wearables for wildlife’, miniature on-animal sensors, quantifying the health of animals and the surrounding environment on the move, and transmitting artificially intelligent summaries of these data globally. We introduce a new class of Internet-of-things (IoT) waveforms—the random-access, very-low-power, wide-area networks (RA-vLPWANs) which enable uncoordinated multiple access at very-low-signal power and low-signal-to-noise ratios. RA-vLPWANs used in ICARUS solve the problems hampering conventional low-power wide area network (LPWAN) IoT systems when applied to space communications. Prominent LPWANs are LoRA, SigFox, MIOTY, ESSA, NB-IoT (5G), or SCADA. Hardware and antenna aspects in the ground and the space segment are given to explain practical system constraints.

## 1. Introduction

The ICARUS satellite IoT system attaches mini-transmitters, called “tags”, to a variety of animal species [1,2]. These tags then send their measurement data, e.g., GPS coordinates, sensor information, or AI-determined patterns of animal behavior, such as sickness or stress, to the ICARUS receiver station, currently operated on the international space station, ISS. The ICARUS receiver station in turn transmits the data to a ground station from where it is sent to the relevant teams of researchers. Collected tag data will finally be archived, distributed, shared, and published through the Movebank database [3] that is accessible to the public. As a spaceborne IoT system, ICARUS aims at data rates less than 1 kbit/s and faces three major technical challenges:

(I) The tag transmit power is low to save battery lifetime. Hence, the signal-to-noise ratio (SNR) of the received signals will be in the order of −30 dB up to −20 dB.
(II) The medium access of all ICARUS tags in range shall be uncoordinated while providing sufficient orthogonality among the tag signals for reception.
(III) Due to the motion of the space platform, the IoT radio signal will exhibit a high and unpredictable Doppler shift as well as considerable multipath propagation.

**Figure 1:**
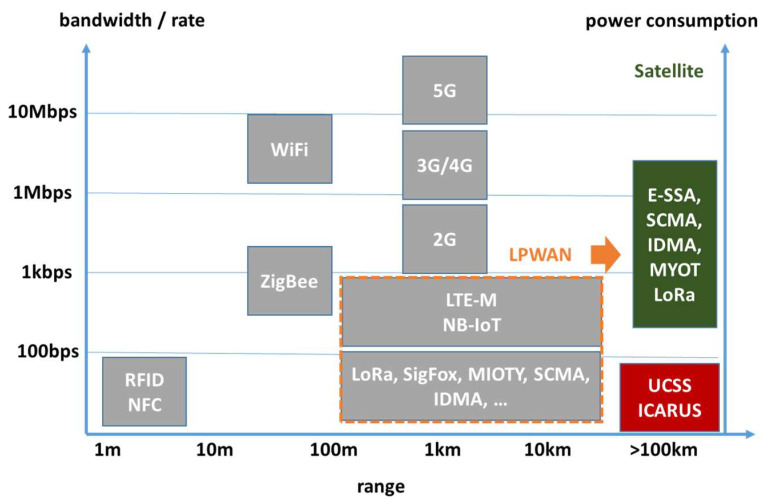
Taxonomy overview of satellite and non-satellite IoT systems.

To cope with these challenges, we introduce a system design entitled random-access, very-low-power, wide-area network, RA-vLPWAN. Besides ICARUS, other RA-vLPWAN systems are known, such as the unipolar-coded chirp spread spectrum (UCSS) system described in [4,5], which exhibits comparable RA-vLPWAN attributes as the ICARUS signaling concept but was made for geostationary satellite communications. Both ICARUS and UCSS are explicitly made to tackle the problems of conventional IoT systems when applied to space communications. These conventional IoT systems are namely LoRA [6], SigFox, MIOTY, ESSA, NB-IoT (5G), and SCADA networks [4,5], which constitute the group of low-power, wide-area networks (LPWANs). Conventional LPWAN systems are made for data rates between some 100 bit/s up to several kbit/s as depicted in Figure 1. Hofmann et al. have shown in [4,5] that other than the newly introduced RA-vLPWAN class, LPWANs cannot be scaled down to lower data rates or to a signal-to-noise ratio as low as expected in space communications for the following reasons:

- LPWAN systems need a minimum data rate of approximately 1 kbit/s to establish stable time and frequency synchronization. These systems are not made for establishing a stable signal synchronization for SNR < −20 dB and high random Doppler shifts.
- Efficiency: All the conventional LPWAN systems use fixed preamble/pilot signal sequences for synchronization. At a very-low-data rate, the spectral efficiency gets prohibitively low since the ratio between pilot and payload data gets worse.
- Some of the well-established IoT systems, such as LoRA, do not support uncoordinated random-access schemes where all tags start transmission at arbitrarily chosen time instants. As a consequence, the number of supported users per satellite beam is very limited and can be, in a worst case, as low as one.
- By design, LPWAN systems, such as NB-IoT, typically are not resilient against huge random Doppler shifts since they are made for terrestrial mobile communications.

Motivated by initial channel measurements in 2015 [7], ICARUS has been the first waveform ever since to introduce the RA-vLPWAN class of IoT systems, tackling all problems of conventional IoT systems when applied to space applications. ICARUS uses uncoordinated random access and allows stable time and frequency synchronization even in low SNR, at low-data rates, and high-Doppler shifts.

The ICARUS system is often compared to the ARGOS satellite-based object-tracking system [8]. However, ARGOS requires a higher UL-signal power and does not provide a backward channel to adjust/trigger specific tag behavior. The ICARUS system can selectively change the behavior (e.g., tag sensor duty cycle) of single tags by using a dedicated downlink control channel from the ISS to ground.

The paper is structured as follows. In Section 2, we explain the ICARUS system concept, and we list all ICARUS signal parameters for the uplink (UL) and the downlink (DL). In Section 3, the properties of the ICARUS radio wave propagation channel are explained, which are important for the digital receiver design. Section 4 provides overview information on the ICARUS tags, where Section 5 will detail link budget parameters and will explain the UL ISS antenna concept. This article closes with Section 6, where first animal behavioral research results are summarized. The main contribution of the paper is twofold: (I) we introduce major technical parameters and design aspects of the ICARUS system; (II) we explicitly explain how the joint time and frequency signal synchronization is realized by means of conjugate complex CAZAC preamble sequences.

## 2. ICARUS System Overview

ICARUS implements an uplink (UL) and a downlink (DL). The uplink is used by the tags to transmit sensor data to the ISS. After tag data reception, the downlink is used to optionally sent re-configuration commands to the tags. The downlink signal is a constant carrier which contains spread QPSK symbols. This continuous carrier is used by the tag as a beacon signal to coordinate wake-up cycles and for timing synchronization. The following Table 1 summarizes the basic signal and data rate properties of both uplink and downlink.

**Figure 2:**
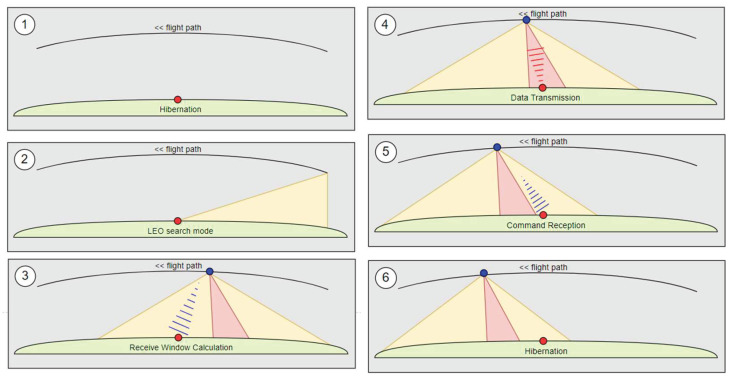
Tag wake-up procedure during ISS overflight: hibernation phase (1), satellite search phase (2), downlink beacon reception (3), uplink window (4), downlink tag command reception (5), orbit propagation for next overflight and go back to hibernation (6).

ISS overflights are used by the tags to transmit one single uplink burst of 223 bytes payload data. Prior to transmission a complex wake-up procedure is realized, which saves battery lifetime. This procedure consists of six individual steps which are explained in Figure 2. During the wake-up and data transmission phases, the tag performs an ISS orbit propagation based on ephemeris data received in the downlink. Based on the tag GPS position and the ISS orbit data, the exact data transmission time instant is calculated to meet the uplink time window of 8 s duration only. This appears quite challenging when considering an uplink burst duration of almost 3.5 s.

## 3. Physical Layer and Doppler Compensation

### 3.1. PHY Overview

**Figure 3:**
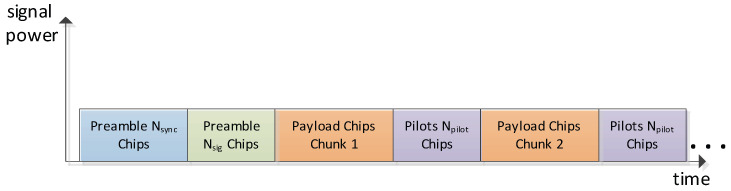
ICARUS uplink bust structure.

ICARUS signal transmission is based on single CDMA (code division multiple access) bursts which are emitted during the ISS overflight. The burst structure is depicted in Figure 3. The preamble is used for time and frequency synchronization. It is explicitly designed to jointly estimate exact burst start timing under high-Doppler shifts and low SNR [9].

**Figure 4:**
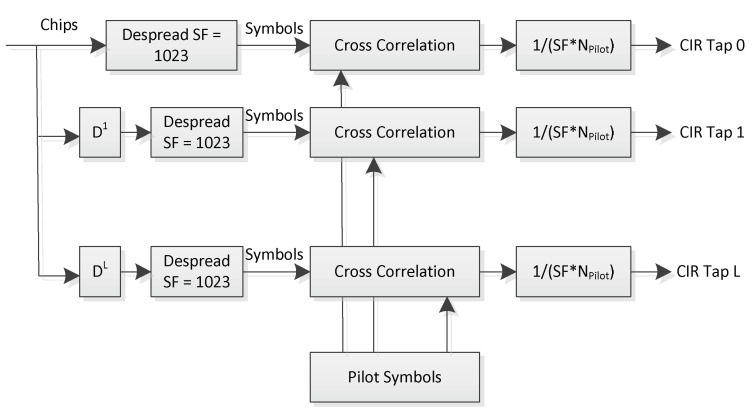
Correlation-based channel estimation.

**Figure 5:**
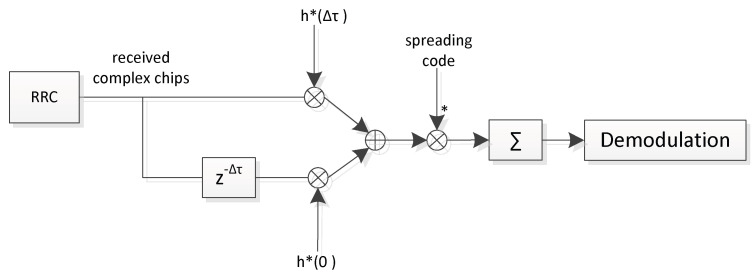
Rake receiver with two rake fingers for channel equalization.

After the synchronization preamble, the signaling preamble (green part in Figure 3) is transmitted which encodes the spreading code ID used for direct-sequence spreading of payload and pilot symbols. The subsequent burst structure then uses an interleaved payload–pilot pattern whereas the pilot symbols are BPSK (binary phase shift keying) encoded, while the payload symbols are QPSK (quadrature phase shift keying). Both payload and pilot symbols are spread with the same spreading code of length 1023. The interleaved pilots are explicitly made for keeping track of the time-varying radio channel impulse response which exhibits volatile multi-path [7]. Channel impulse response (CIR) estimation is performed using the inserted pilot sequences to constantly track the Rake receiver [10] which is used to equalize and to de-spread the received CDMA chips. Figure 4 shows the architecture of the channel estimation circuitry used in the ISS spaceborne receiver:

The principle of the ICARUS Rake receiver is depicted in Figure 5 for the case where two so called ‘Rake fingers’ are used. Each Rake finger corresponds to one single channel tap, all of which are equalized by a maximum-ratio-combiner. Figure 5 shows the schematics of the implemented Rake receiver for two rake fingers, whereas ten Rake fingers are used in the uplink space-borne receiver, and five fingers are used in the ICARUS tag to equalize the downlink signal. The * sign in Figure 5 denotes conjugate complex signal operation.

The rake receiver performs constructive signal combining by means of a maximum ratio combining operation [10]. It exploits the good auto-correlation properties of the spreading code which nearly cancels out time-shifted versions of the received signal. As an important side effect, the Rake receiver is allowed to keep signal orthogonality of different tag signals even after equalization in low SNR (<−20 dB). It is allowed to operate a random access (RA) channel even under difficult channel properties and low SNR. These important properties lead to the definition of the new class of space IoT systems: RA-vLPWAN.

**Figure 6:**
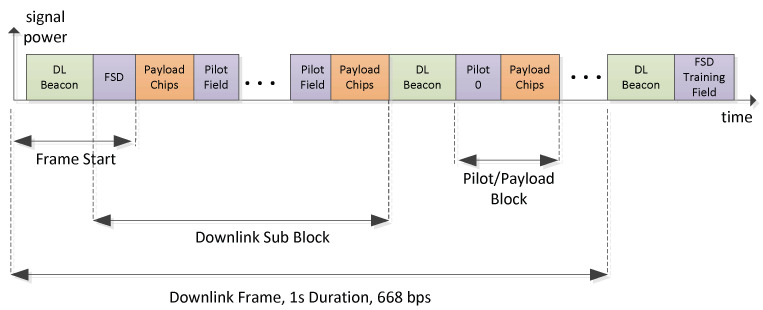
ICARUS downlink burst structure.

The downlink signal structure (see Figure 6) is similar to the uplink burst structure, whereas the DL signal forms a continuous stream of frames, realizing a continuous carrier. The DL beacon has a similar structure as the UL synchronization preamble. It is made for time and frequency synchronization under high-Doppler shift and low SNR. Regular pilot insertions allow it to track the time-varying channel impulse response used for Rake reception. The first pilot field is different from other pilot fields since it implements the frame start delimiter (FSD) which marks the start of a new LDPC FEC frame.

### 3.2. Joint Timing and Frequency Synchronization

The joint time and frequency synchronization is difficult under the presence of large random Doppler shifts at low SNR. Conventional cross-correlation-based time and frequency synchronization typically uses pseudo-noise bipolar sequences, so called M-sequences [9,11]. In ICARUS, we use a CAZAC (constant amplitude zero auto-correlation) sequence. Both sequence type options are compared in the following Table 2.

When using bipolar M-sequences the correlation peak amplitude strongly depends on the Doppler shift of the received signal. The Doppler shift introduces a carrier frequency offset (CFO) of the received signal to its nominal center frequency which causes significant degradations.

**Figure 7:**
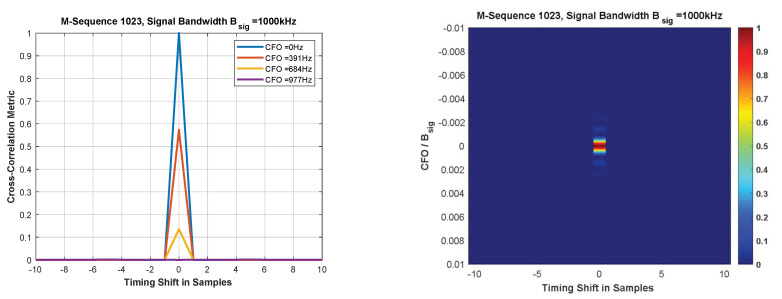
Cross-correlation performance of bipolar M-sequences used for time–frequency synchronization.

Figure 7 shows the cross-correlation peak degradation at high-carrier frequency offset values which makes M-sequences unsuitable for space IoT systems when operated with LEO (low earth orbit) satellite systems. When using CAZAC sequences, the performance changes [11,12].

**Figure 8:**
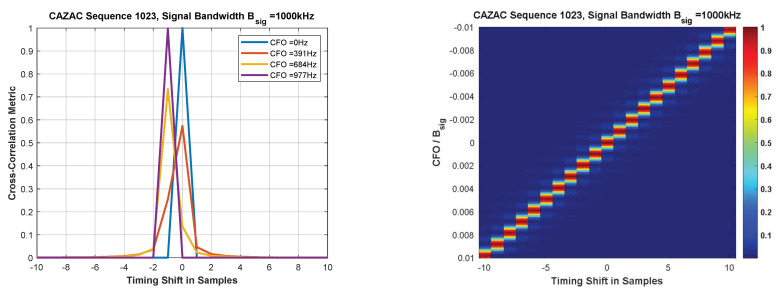
Cross-correlation performance of a single CAZAC sequence used for time–frequency synchronization.

Figure 8 exhibits a preferable immunity of the cross-correlation peak amplitude against CFO. However, we observe a linear timing lag of the peak versus CFO. The linear timing lag appears quite impractical in the uplink when considering random Doppler shifts of the incoming tag signals.

The ICARUS solution to jointly estimate timing and Doppler shift is to use a form of CAZAC sequences, widely known as Frank–Zhadoff–Chu (FZC) sequences. The UL and DL synchronization preamble sequences are composed of two consecutive complementary FZC subsequences. Subsequences 1 and 2 are defined as follows where the ICARUS parameters are listed in Table 3.

(1) $$z_{\mathrm{FZC}}\left(n\right)=\;\exp\left(\mathrm{jd}\pi \frac{\mathrm{un}\left(1+n+2q\right)}{N_{Z}}\;\right)$$

where $N_{Z}$ denotes the sequence length. Variable n denotes the sample time domain index ranging from [0, ..., $N_{Z}$−1]. Value d is either set to 1 or −1, where u and q are carefully chosen signed integer values.

**Figure 9:**
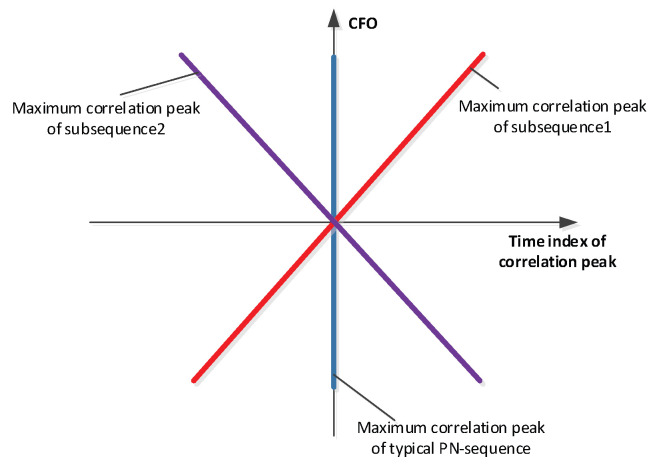
Subsequence ambiguity in time and frequency shift.

**Figure 10:**
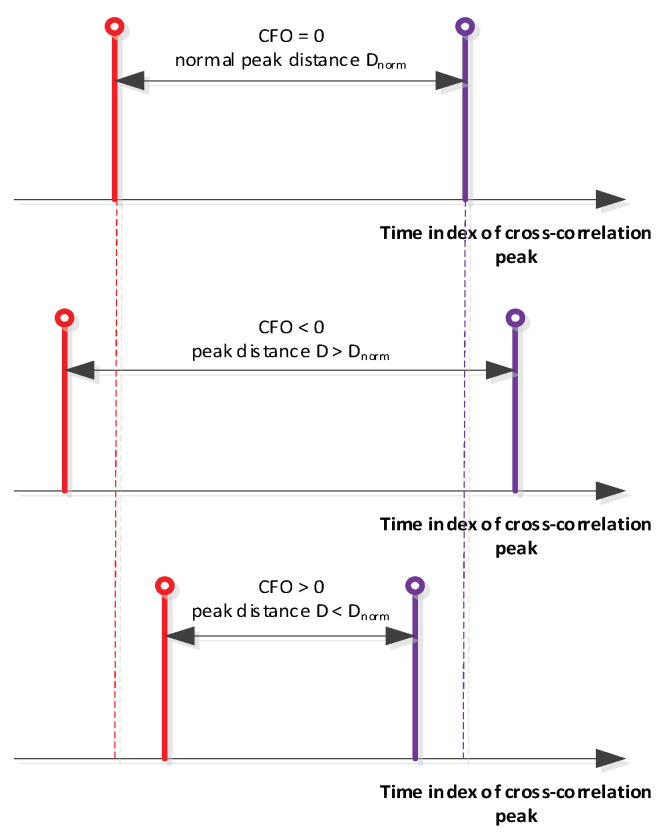
Algorithmic idea of ICARUS joint timing and Doppler sync.

The benefit of using two FZC sequences that are conjugate complex copies of each other is the symmetric timing shift property depicted in Figure 9. The correlation peak timing lag of both subsequences is inverted equal at a given CFO value. With this property in mind, an easy CFO and timing algorithm could be generated which allows the ICARUS system to be jointly synchronized in time and frequency. This synchronization algorithm is described in Figure 10. 

After the first correlation peak (subsequence 1) is detected, the receiver cross-correlator switches to subsequence 2. When detecting the cross-correlation peak of subsequence 2, the timing difference of both peaks is measured. Due to the properties of the FZC sequence, the timing difference of both peaks is directly proportional to the CFO/Doppler shift which allows it to generate a simple look-up table approach to estimate timing and CFO. A similar strategy of CFO estimation has been used in [5].

The challenge of using FZC sequences lies in its elements which are equally spread among the unit circle of the complex number plane. In practice, it appears too complex to store these complex numbers in a ROM look-up table (LUT) with low bit-width. 

**Figure 11:**
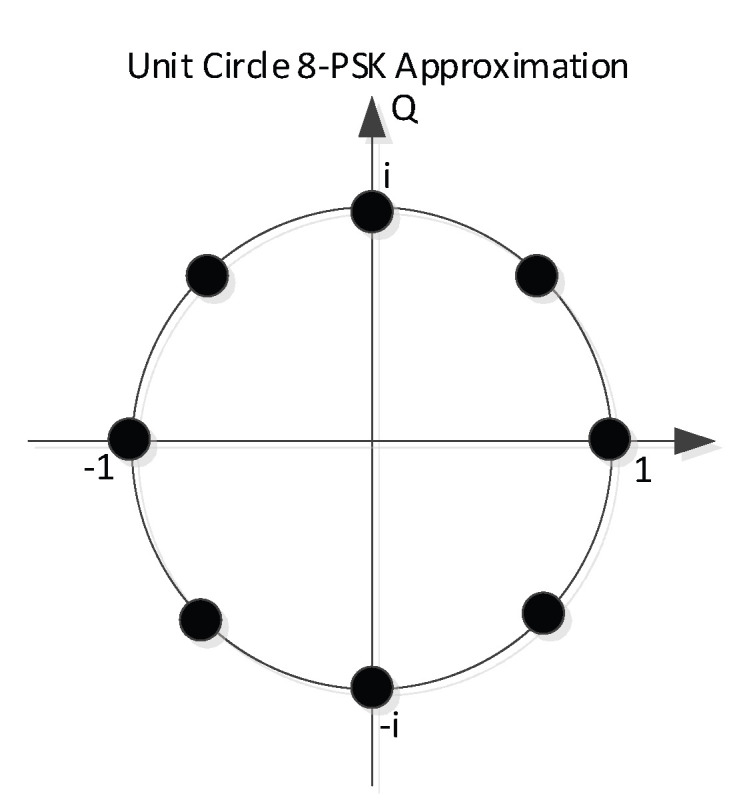
8-PSK approximation of the unit-circle FZC baseband symbols.

Instead, it appeared beneficial to use unit circle approximations of the original FZC subsequences. In ICARUS we hence applied an 8-PSK mapping of the FZC sequence, as depicted in Figure 11. 

Each element of subsequences 1 and 2 are rounded towards one of the eight elements of the given 8-PSK constellation. System simulations showed only a negligible performance degradation when compared with the full-circle FZC implementation but saves LUT space since only three bits need to be stored per sequence element. Complex multiplication of 8-PSK constellations is easier to implement in FPGA logic compared to high-resolution complex multipliers which is also of great importance with respect to the real-time implementation on ISS.

### 3.3. ICARUS Channel Properties

#### 3.3.1. Doppler Effect

**Figure 12:**
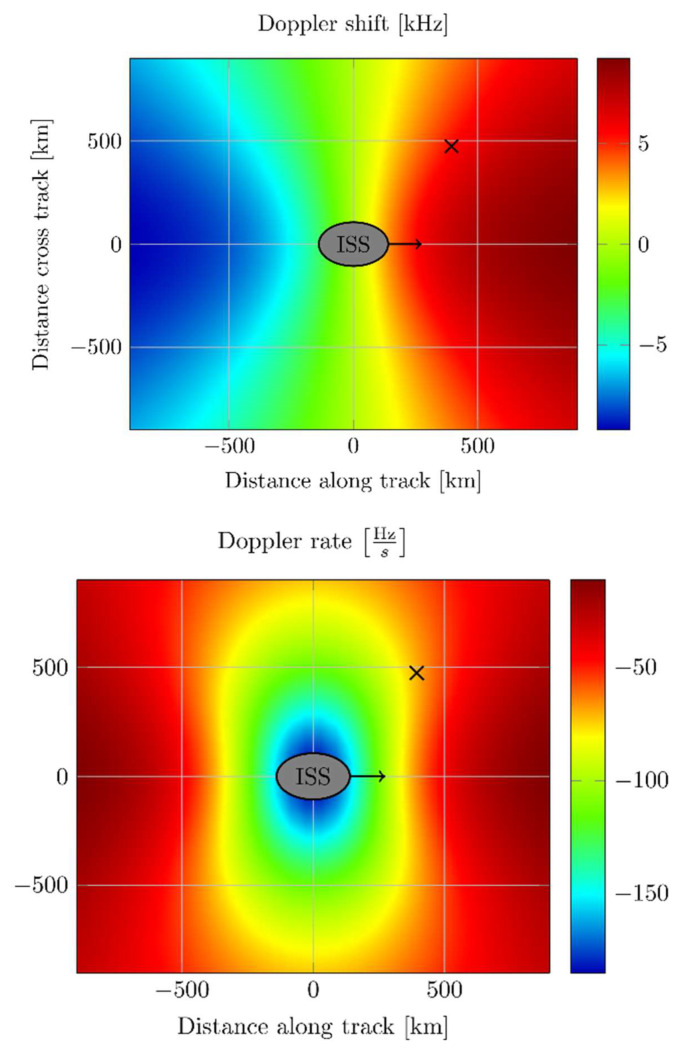
Doppler parameter of ICARUS.

As the ISS travels at a high velocity, the communication links are influenced by the Doppler effect. The Doppler effect is mainly characterized by the well-known Doppler frequency shift which imposes a time-varying carrier frequency offset on the received signal. The first derivative of the Doppler shift is of importance as well. It is denoted as Doppler rate [Hz/s] which describes the change of the frequency offset over time. Figure 12 outlines the main characteristics of the Doppler shift and the Doppler rate along ground track of the ISS.

#### 3.3.2. Radio Channel Properties and Propagation Conditions

**Figure 13:**
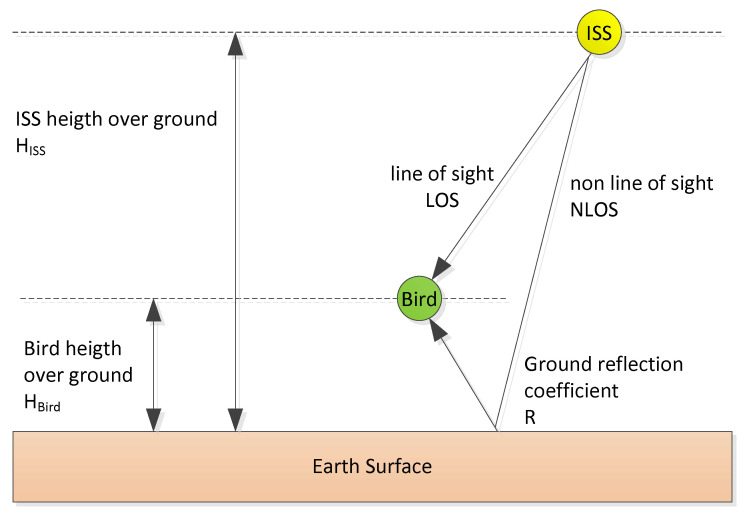
ICARUS Multipath Propagation Scenario.

Much effort have been made to properly understand the ICARUS propagation channel at the selected transmission frequencies [7] before designing the physical layer of our transmission system. Figure 13 depicts the typical ICARUS communication signal propagation scenario which is modelled as a classical 2-ray channel model. Both signal components dominate the entire propagation setup: the direct path (LOS—line of sight) and the reflection paths (NLOS—non-line of sight). A dedicated additional path attenuation of the reflection component is introduced by means of the ground reflection coefficient, R.

As a reference for multiple ground types, Table 4 lists reflection coefficients, as outlined in [13].

**Figure 14:**
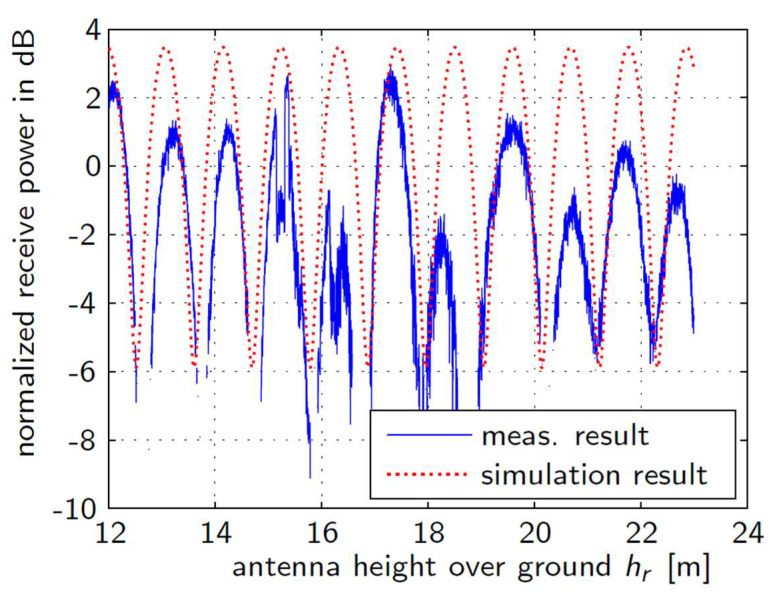
ICARUS Multipath Propagation Scenario.

Figure 14 shows measured normalized receive signal power of the ICARUS downlink with vertical polarized antenna versus height of the receiver over ground. We compare the measurement results with the simulation result for the two-ray model with a ground reflection on wet ground.

**Figure 15:**
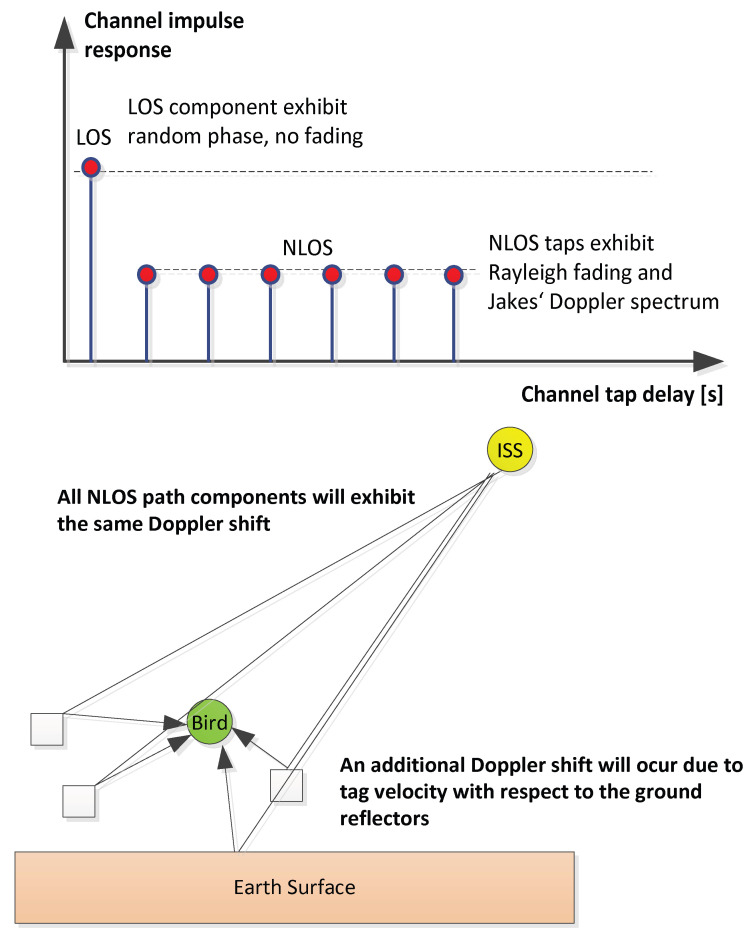
ICARUS channel impulse response modelling.

Any variation of the antenna height causes an alternation between constructive and destructive superposition of the line-of-sight signal and the ground reflection signal. The simulation result from the two-ray model coincides well with the measured data with respect to the deep fades of the signal power. There might however be propagation scenarios which are not dominated by a non-resolvable ground reflection. In such cases, we need to model the radio channel as a complex base-band impulse response which exhibits resolvable LOS and NLOS tap components, as depicted in Figure 15.

**Figure 16:**
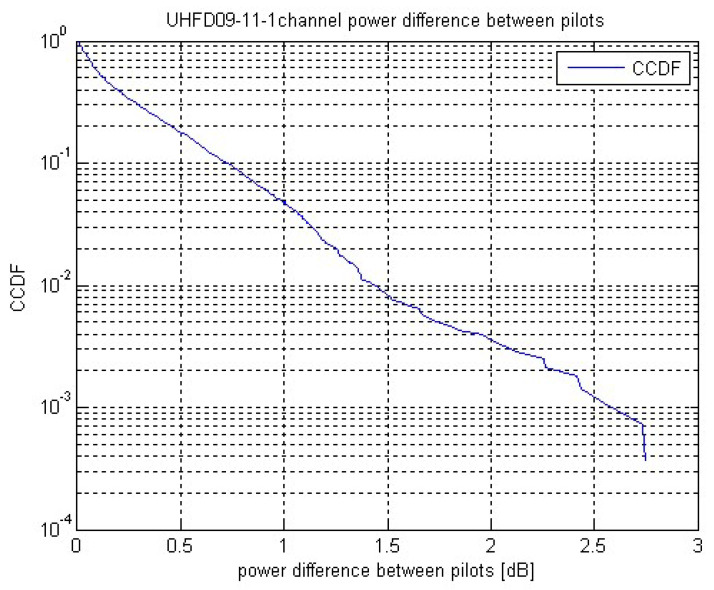
CCDF of inter-pilot channel power difference.

The LOS component is modelled to be a slowly time-varying channel tap of random phase. The NLOS taps are modelled to be mutually independent Rayleigh faded random variables. The ISS velocity causes a time-varying radio channel which is characterized by receive power fluctuations during UL or DL packet reception. The downlink signal power deviations between consecutive pilot blocks is exemplarily and statistically evaluated in Figure 16 by means of its CCDF (complementary cumulative distribution function).

Based on the channel measurement campaigns and the ICARUS receiver implementations, we can derive the following implications on the ICARUS system design:

1. UL: The appliance of a bit interleaver prior to FEC encoding [14] shows negligible impact on system performance. This is because the UL frame is QPSK-modulated and FEC-encoded by a strong LDPC code.
2. The ICARUS UL performance is dominated by long-term fades due to ground reflection; fast fading does not show any considerable performance impact since it can be tracked easily by the inserted pilot signal sequences.
3. The current UL and DL pilot distance setting appears to be chosen right.
4. The current receiver implementation can cope with strongly faded pilot blocks if the fading duration is no longer than two pilot blocks (no corruption of timing and CFO tracking).

## 4. The ICARUS Tag

**Figure 17:**
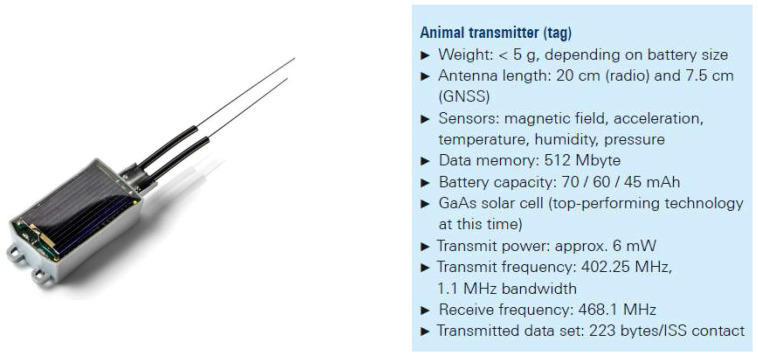
ICARUS tag overview.

The most important condition for a viable animal transmitter (tag) is that the species under study must tolerate wearing the tag as a true ‘wearable for wildlife’. As recommended by ethics committees, the tag should not exceed 3% of the animal’s body weight to avoid influencing the animal’s behavior or even endangering it. Since it was also planned to equip small animals with the tags, the upper limits for the size and weight were very difficult to meet. The trackers based on mobile or analog radio, which were conventional at that time, were ruled out for animals under 1 kg, meaning that 75% of all bird and mammal species could not be studied. The blackbird was chosen by the MPIAB as a reference animal due to the long-term focus on this songbird within the observation program. Prototypes of the ICARUS tag were tested, preferably on these seemingly familiar birds whose migration behavior still brings up questions, though that could only be answered by means of continuous monitoring. Weighing in at 4.5 g, the lightest version of the tag is just light enough for the blackbird, assuming its use is limited to adult male specimens (see Figure 17). For all other species to be fitted with a transmitter according to current ICARUS planning, the recommendation is easily fulfilled.

Along with the radio and location technology, the tags contain multiple sensors as well as enough memory to store the movements and environmental data for a single animal during its entire life. Up to 20 sets of position data are transmitted to the ISS during each overhead pass, which generally occurs daily but can take place every three days at lower, equatorial latitudes. The limited amount of data is due to the brief contact window of only 8 s (of which 3.4 s are used for transmission) and the low bandwidth and high-spreading factor of the UL signal. The fact that a miniature radio with only six milliwatts of transmit power can communicate with a satellite is an important property of this RA-vLPWAN system. The tags use the regularly transmitted ISS ephemeris data along with their own position to calculate the next time of contact. They prepare to receive and transmit during the calculated time window, but mostly remain in stand-by mode to save power. Based on the regularly transmitted tracking data that is compiled in the database at movebank.org [3], researchers have already gained valuable insights (see Section 6). However, another component is needed to access the entire data trove accumulated by the tags, including the environmental data.

## 5. ISS Antenna Concept and Link Budget

**Figure 18:**
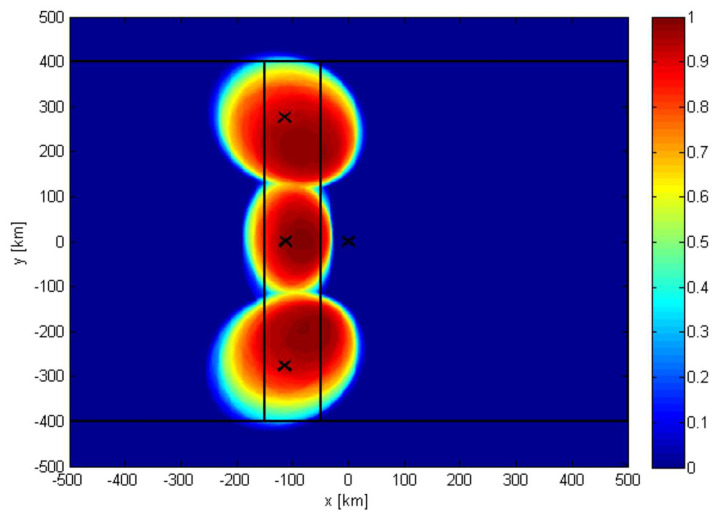
ICARUS uplink satellite antenna gain contour, color code: probability of successful packet reception.

The ICARUS packet reception performance strongly depends on the UL and DL link budget. The UL link budget is especially crucial for gaining the tag position data. The following Table 5 hence summarizes the major parts of the ICARUS UL link budget:

The ICARUS UL antenna consists of three independent antenna elements which are slightly steered in the backward direction (relative to ISS orbit path). Two of the antenna elements are steered off-track. Each of the antenna elements feeds a separate digital receiver branch which increases demodulation capacity. The following Figure 18 shows the UL satellite antenna gain contours as a projection on ground. The color code indicates the probability of successful UL packet reception depending on random channel fading and the given link budget.

## 6. ICARUS Research on Animal Behavior

**Figure 19:**
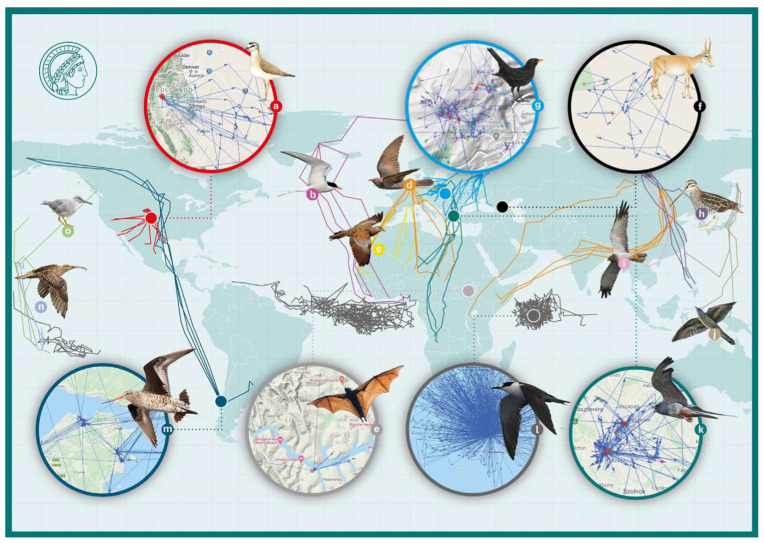
Overview of first ICARUS data. The global map displays large-scale tracks of selected individuals of 15 species (a–o); the inset maps show regional-scale tracks of seven species during this period. Map redrawn from Jetz et al. 2022 [1], with permission.

Despite the substantial problems of sending data from a mobile animal, the ICARUS tags performed extremely well all around the globe [1]. We received data from all latitudes and longitudes within the orbit of the ISS (ca. 56 degrees), as well as archived GPS positions from animals that temporarily went outside (north or south of) the orbit of the ISS (see Figure 19), i.e., could not be read out by the antenna and receiver on the ISS for some time. Transmissions occurred from inside the rain forest, through heavy weather systems (thunderstorms, typhoons, etc.), in dry and hot areas (e.g., Sahara Desert), and from all oceans [15].

During the first year of operation (ca. 2021 March–2022 March), we received valid payload data for 3090 individual ICARUS tags via the ISS. This represents a total of 62,791 individual valid ISS payload data (contacts to ISS). A total of 4 GB of payload data in 979 files was transferred to the ICARUS User Data Center and from there, directly into the global database, Movebank [16]. Within the database, Movebank, ICARUS hosts 2281 deployments of tags registered on individual animals. The global collaboration partners within the ICARUS initiative studied a total of 75 different animal species. In total, these deployments of tags on animals include 168,913 GPS fixes transmitted through space to the ISS. In addition, the ICARUS tags can be read out terrestrially via a handheld receiver. Here, more data can be transmitted, and thus, the total number of GPS fixes from ICARUS tags on animals in Movebank is substantially higher [17].

Scientific highlights and transformations in our knowledge about animal movements are, e.g., provided by the tracking of common cuckoos from Kamchatka to Southern Africa, the concurrent tracking of seabirds, such as Sooty terns in three oceans of the world (Atlantic Ocean: Asencion Island; Indian Ocean: Seychelles; Pacific Ocean: Polynesia) as sentinels of climate change and typhoon initiation, the first return migration tracks of European blackbirds, the tracking of African fruit bats to understand their role as ecosystem service agents and their sentinel function to find where the true host of the Ebola virus hides, or the year-round study of endangered mountain plovers in the Rocky Mountains of the USA [15,18].

The excellent technical performance of the ICARUS tracking and communication system has started to allow us to address scientific questions in biology and ecology that were completely out of reach even 1.5 decades ago [18,19,20].

## Figures and Tables

**Table 1:** ICARUS Uplink and Downlink System Parameters.

	Uplink (UL)	Downlink (DL)
Carrier	burst traffic, one single burst per tag per ISS overflight, multiple access channel	Constant carrier used to synchronize tags and to broadcast tag-individual commands, broadcast channel
Modulation	QPSK modulated CDMA, Spreading factor 1023	QPSK modulated DS-Spread Spectrum, Spreading Factor 23
Chip rate	900 kHz	33.75 kHz
Roll-Off	30%	30%
Center frequency	402.25 MHz	468.1 MHz
Net data rate after FEC decoding and overhead	560 bit/s, 223 Byte/1784 Bit per uplink burst	668 bit/s
FEC	irregular-repeat-accumulate LDPC rate 0.4	irregular-repeat-accumulate LDPC rate 0.4667
Number of Rake fingers	10	5
Tolerable multi-path	11 µs (3.3 km)	148 µs (44 km)
Typical SNR	−30 dB up to −20 dB	−5 dB up to 5 dB

**Table 2:** Design Tradeoffs for Different Preamble Types.

	M-Sequences (Bipolar)	CAZAC (Complex Sequences)
Timing offset estimation accuracy	High	Average
Frequency offset tolerance	Low	High
Cross-correlator complexity	Low, binary sequences do not require MULT operations during correlation	High, complex sequences require complex MULT operations during correlation
Signal generation	Easy (bipolar BPSK type sequence)	Average (complex valued IQ sequence)

**Table 3:** Downlink Beacon Sync Fields.

FZC Parameter	Subsequence 1	Subsequence 2
d	−1	1
u	1	1
q	0	0
Nz Downlink	255 = 2^8^ − 1	255 = 2^8^ − 1
Nz Uplink	16,383 = 2^14^ − 1	16,383 = 2^14^ − 1

**Table 4:** Reflection Coefficients for Typical Ground Types.

Surface	Dielectric Constant ε_r_	Reflection Coefficient R
Dry poor ground	4–7	−9.7 dB, ..., –7.0 dB
Average ground	15	−4.7 dB
Wet ground	25–30	−3.6 dB, ..., −3.3 dB
Water	81	−1.97 dB

**Table 5:** ICARUS UL Link Budget.

Parameter	Value in 402.25 MHz
Chip rate	900 kHz
Spreading factor	1023
Orbit height	400 km (time varying)
Max. single-sided UL antenna opening angle	55°
Max. Slant path (direction edge of coverage)	872 km
Swath width	800 km depending on ISS orbit height
UL tag EIRP	8 dBm (derived from tag antenna chamber measurement campaigns)
UL tag antenna gain due to imperfect pointing	−1 dBi
Satellite Rx antenna gain max	4 dBi in bore sight (3 antenna elements each of a different backward tilt and off-track directivity)
Satellite Rx antenna gain edge of coverage	1 dBi
Free space loss to Nadir	−138.5 dB
Free space loss to edge of coverage	−143.3 dB
Atmospheric losses	0.1 dB
Fading loss	0 dB (Rayleigh fading will be simulated in link budget calculations)
Satellite Rx feeder loss	0.5 dB
Satellite antenna noise temperature including man-made interference	2000 K (worst case), 700 K average case
Satellite receiver temperature	500 K
Polarization loss	3 dB
